# The Effect of Etoricoxib on Hepatic Ischemia-Reperfusion Injury in Rats

**DOI:** 10.1155/2015/598162

**Published:** 2015-07-05

**Authors:** Celalettin Semih Kunak, Osman Kukula, Emre Mutlu, Fatma Genç, Gülçer Güleç Peker, Ufuk Kuyrukluyıldız, Orhan Binici, Durdu Altuner, Hamit Hakan Alp

**Affiliations:** ^1^Department of Pharmacology, Faculty of Medicine, Ordu University, Ordu, Turkey; ^2^Department of Pharmacology, Faculty of Medicine, Ondokuz Mayıs University, Samsun, Turkey; ^3^Department of Pharmacology Nursery, Faculty of Health Science, Giresun University, Giresun, Turkey; ^4^Department of Medical Device and Drug Institute, Turkish Health Ministry, Ankara, Turkey; ^5^Department of Anesthesiology and Reanimation, Faculty of Medicine, Erzincan University, 24030 Erzincan, Turkey; ^6^Department of Pharmacology, Faculty of Medicine, Erzincan University, 24030 Erzincan, Turkey; ^7^Department of Biochemistry, Faculty of Medicine, 100 Yıl University, Van, Turkey

## Abstract

Ischemia-reperfusion (I/R) damage is known to be a pathological process which continues with the increase of oxidants and expands with the inflammatory response. There is not any study about protective effect of etoricoxib on the liver I/R damage in literature. *Objective*. This study investigates the effect of etoricoxib on oxidative stress induced by I/R of the rat liver. *Material and Methods*. Experimental animals were divided into four groups as liver I/R control (LIRC), 50 mg/kg etoricoxib + liver I/R (ETO-50), 100 mg/kg etoricoxib + liver I/R (ETO-100), and healthy group (HG). ETO-50 and ETO-100 groups were administered etoricoxib, while LIRC and HG groups were orally given distilled water by gavage. Hepatic artery was clamped for one hour to provide ischemia, and then reperfusion was provided for 6 hours. Oxidant, antioxidant, and COX-2 gene expressions were studied in the liver tissues. ALT and AST were measured. *Results*. Etoricoxib in 50 and 100 mg/kg doses changed the levels of oxidant/antioxidant parameters such as MDA, MPO, tGSH, GSHRd, GST, SOD, NO, and 8-OH/Gua in favour of antioxidants. Furthermore, etoricoxib prevented increase of COX-2 gene expression and ALT and AST levels. This important protective effect of etoricoxib on the rat liver I/R can be tested in the clinical setting.

## 1. Introduction

As is known, ischemia is a phenomenon of restriction or complete cease in arterial or venous blood supply to tissues, causing a shortage of oxygen. Reperfusion is returning of blood supply to the tissues after a period of ischemia. Ischemia-reperfusion (I/R) process applied during transplantation and resection of the liver is the most important cause of cellular death and liver dysfunction. Therefore, I/R causes serious problems [1]. Even so, reperfusion process leads to much more severe damage compared to that caused by ischemia [2]. Recent studies proposed that I/R damage is a complex pathological process which begins with lack of oxygen in tissues, continues with change of the oxidant/antioxidant balance in favor of oxidants, and expands with inflammatory response [3]. Vascular endothelial cells activated by I/R produce free oxygen radicals (ROSs) in more abundant amounts. This phenomenon leads to the production and releasing of inflammatory mediators; excessive formation of free radicals in I/R damage causes activation of polymorphonuclear leukocytes (PNL), further increasing production of ROSs [4, 5]. In turn, excessively produced free oxygen radicals defeat oxidation on cellular membrane lipids, leading to formation of toxic products such as malondialdehyde (MDA) from lipids. In addition, oxygen radicals also react with DNA, causing oxidative damage; 8-hydroxyguanine (8-OHGua) is a product of oxidative DNA damage [6]. Demiryilmaz et al. reported that levels of MDA, myeloperoxidase (MPO), 8-OH/Gua, and cyclooxygenase-2 (COX-2) increased, while levels of glutathione (GSH) decreased in the liver tissue which has been subjected to I/R [7]. Again it was reported in another study that there was a direct correlation between the altered COX-1/COX-2 activities and oxidant/antioxidant balance in I/R damage [3]. This information from the literature indicates that selective COX-2 inhibitors which have antioxidant feature may play a role in prevention or attenuation of the liver I/R damage.

In this study, etoricoxib, a COX-2 selective inhibitor, was used to test its effects on liver I/R injuries [8, 9]. Etoricoxib has been reported to suppress oxidative stress due to experimental renal I/R [3]. Again, in a recent study, etoricoxib has been demonstrated to significantly prevent increasing of COX-2, MDA, and MPO and decreasing of HSH and COX-1 in the ovarian tissue subjected to I/R [10]. This information suggests that etoricoxib may be beneficial in prevention or treatment of the damage caused by I/R process which is applied in the liver tissues. No information has been found about protective effect of etoricoxib on liver injury induced with ischemia and reperfusion. Therefore, the objective of this study was to investigate the effect of etoricoxib on oxidative stress induced by I/R of the rat liver.

## 2. Material and Methods

### 2.1. Experimental Animals

Experimental animals were obtained from the Recep Tayyip Erdoğan University, Medical Experimental Research and Application Center. A total of 24 male Wistar rats weighed between 230 and 240 g were randomly selected for use in the experiment. The animals were housed and fed in the pharmacology laboratory at normal room temperature (22°C) for one week before the experiment in order to provide adaptation to their environment. Animal experiments were performed in accordance with the National Guidelines for the Use and Care of Laboratory Animals and were approved by the local animal ethics committee of Recep Tayyip Erdoğan University, Rize, Turkey (Ethics Committee number: 2014/68, dated: 30/10/2014).

### 2.2. Chemicals

Etoricoxib used in the experiment was supplied from Merck Sharp & Dohme, England, and Thiopental Na was supplied from İE ULAGAY (Türkiye).

### 2.3. Experiment Groups

Experimental animals were divided into four groups as liver I/R control (LIRC), 50 mg/kg etoricoxib + liver I/R (ETO-50), 100 mg/kg etoricoxib + liver I/R (ETO-100), and healthy group sham operated (HG).

### 2.4. Anesthesia Procedure

Surgical procedures on rats were performed under sterile conditions by administration of 25 mg/kg thiopental sodium intraperitoneally. After thiopental sodium injection, the rats were waited until an appropriate period appeared for the surgical intervention. Period during which the rats were inactive was considered as the appropriate anesthesia period [7].

### 2.5. Pharmacological and Surgical Procedures

One hour before thiopental sodium anesthesia, ETO-50 group was orally administered 50 mg/kg etoricoxib and ETO-100 group 100 mg/kg oral etoricoxib by gavage, whereas LIRC and HG rat groups were given distilled water as the solvent by the same method. Laparotomy was performed in anterior part of the abdomen by vertically opening 3.5–4 cm long in the anesthetized rats. Then, hepatic artery was clamped (except HG group) in order to create total hepatic ischemia, providing one-hour ischemia and 6-hour reperfusion. At the end of this duration, the rats groups were killed by high doses of anesthesia and levels of oxidant/antioxidant parameters such as MDA, MPO, tGSH, GSHRd, GST, SOD, NO and 8-OH/Gua, and COX-2 gene expression in the liver tissues were determined. Blood values of ALT and AST were measured. Results obtained from the ETO-50 and ETO-100 groups were evaluated in comparison with those of the LIRC and HG groups and evaluated.

## 3. Biochemical Procedures

### 3.1. Preparation of Samples

#### 3.1.1. Determination of Myeloperoxidase (MPO) Activity

In order to determine MPO in the liver tissue, 0.5% HDTMAB (hexadecyl trimethyl ammonium bromide) containing potassium phosphate buffer at pH = 6 and for determination of MDA 1.15% potassium chloride solution and for the other measurements in phosphate buffer at pH = 7.5 were completed to 2 mL and homogenized at 4°C. Then the solution was centrifuged 10,000 rpm for 15 min at 4°C and the supernatant was used for the measurement of MPO activity employing standard methods [11].

#### 3.1.2. Malondialdehyde (MDA) Analysis

MDA measurements are based on the method used by Ohkawa et al. [12]. This method is based on spectrophotometrical measurement of absorbance of the pink colored complex which is formed by thiobarbituric acid (TBA) and MDA at a high temperature (95°C), at 532 nm wavelength. Homogenates were centrifuged at 5000 g for 20 minutes and these supernatants were used to determine amount of MDA. Aliquots of 250 *μ*L homogenate, 100 *μ*L 8% sodium dodecyl sulfate (SDS), 750 *μ*L 20% acetic acid, 750 *μ*L 0.08% TBA, and 150 *μ*L purified water were mixed in a capped test tube and vortexed. Following incubation for 60 minutes at 100°C, 2.5 mL n-butanol was added to the mixture and the absorbance was recorded using a spectrophotometer at 532 nm. Amounts of the red color formed were read at 532 nm using cuvettes of 3 mL and, taking account of dilution coefficients, MDA amounts of the samples were determined using the standard chart which was created through the previously prepared MDA stock solution.

#### 3.1.3. Total Glutathione (tGSH) Analysis

The amount of GSH in the total homogenate was measured according to the method of Sedlak and Lindsay with some modifications [13]. The sample was weighed and homogenized in 2 mL of 50 mmol/L Tris–HCl buffer containing 20 mmol/L EDTA and 0.2 mmol/L sucrose at pH 7.5. The homogenate was immediately precipitated with 0.1 mL of 25% trichloroacetic acid, and the precipitate was removed after centrifugation at 4200 rpm for 40 min at 4°C and the supernatant was used to determine GSH level. A total of 1500 *μ*L of measurement buffer (200 mmol/L Tris–HCl buffer containing 0.2 mmol/L EDTA at pH 7.5), 500 *μ*L supernatant, 100 *μ*L DTNB (10 mmol/L), and 7900 *μ*L methanol were added to a tube and vortexed and incubated for 30 min in 37°C. 5,5-Dithiobis(2-nitrobenzoic acid) (DTNB) was used as a chromogen and it formed a yellow-colored complex with sulfhydryl groups. The absorbance was measured at 412 nm using a spectrophotometer (Beckman DU 500, USA). The standard curve was obtained by using reduced glutathione.

#### 3.1.4. Glutathione Reductase (GSHRd) Analysis

GR activity was determined spectrophotometrically by measuring the rate of NADPH oxidation at 340 nm according to Carlberg and Mannervik method [14]. After tissue homogenization, supernatant was used for GR measurement. After the NADPH and GSSG addition, chronometer was on and absorbance was measured for 5 min by 30 min intervals at 340 nm spectrophotometric methods.

#### 3.1.5. Glutathione s-Transferase (GST) Activity

GST activity was determined by Habig and Jakoby [15]. Briefly, the enzyme's activity was assayed spectrophotometrically at 340 nm in a 4 mL cuvette containing 0.1 M PBS (pH 6.5), 30 mM GSH, 30 mM 1-chloro-2,6-dinitrobenzene, and tissue homogenate.

#### 3.1.6. Superoxide Dismutase (SOD) Analysis

Measurements were performed according to the method of Sun et al. [16]. When xanthine is converted into uric acid by xanthine oxidase, SOD forms. If nitro blue tetrazolium (NBT) is added to this reaction, SOD reacts with NBT and a purple-colored formazan dye occurs. The sample was weighed and homogenized in 2 mL of 20 mmol/L phosphate buffer containing 10 mmol/L EDTA at pH 7.8. The sample was centrifuged at 6000 rpm for 10 minutes and then the brilliant supernatant was used as assay sample. The measurement mixture containing 2450 *μ*L measurement mixture (0.3 mmol/L xanthine, 0.6 mmol/L EDTA, 150 *μ*mol/L NBT, 0.4 mol/L Na_2_CO_3_, and 1 g/L bovine serum albumin), 500 *μ*L supernatant, and 50 *μ*L xanthine oxidase (167 U/L) was vortexed. Then it was incubated for 10 min. At the end of the reaction, formazan occurred. The absorbance of the purple-colored formazan was measured at 560 nm. As more of the enzyme exists, the least O_2_^−^ radical that reacts with NBT occurs.

#### 3.1.7. Nitric Oxide Determination

Nitric oxide levels were measured using the Griess reaction, which is based on a two-step process. In the first step, nitrate is converted into nitrite by nitrate reductase. In the second step, nitrite reacts with the Griess reagent. At the end of this reaction, a deep purple azo compound forms. The absorbance of this azo compound was measured photometrically at the 540 nm wavelength. This azo chromophore accurately determines nitrite concentrations as a marker of NO [17, 18].

### 3.2. DNA Oxidation Analysis

#### 3.2.1. Tissue Preparation

The 50 mg tissue was homogenized at +4°C using 1 mL of homogenization buffer solution [30 mM Tris pH 8, 10 mM EDTA, 10 mM 2-mercapto ethanol, and 0.5% (v/v) Triton X-I00 (*Sigma-Aldrich, Germany*)]. The mixture was centrifuged for 10 minutes at 1000 g and the supernatant was discarded. The pellet was resuspended using 1 mL of extraction buffer (0.1 M Tris pH 8, 0.1 M NaCl, and 20 mM EDTA) and was then homogenized using a vortex for 30 seconds. After that, it was centrifuged at 1000 g for 2 minutes. The pellet was again resuspended using the extraction buffer solution. The suspension was mixed well using the vortex. 400 *μ*L of phenol (*Sigma-Aldrich, Germany*) was added to the mixture and it was mixed thoroughly for 1 minute using the vortex. After 10 minutes of waiting for the phases to separate, we removed the upper phase and transferred it to a clean tube. Then, 400 *μ*L of chloroform-isopropanol (*Sigma-Aldrich, Germany*) was added to the clean tube (24 : 1) and it was centrifuged at 10000 ×g for 10 minutes. The upper phase was again transferred into a new tube. Next, 40 *μ*L of 3 M sodium acetate (*Merck, Germany*), pH = 5, and 800 *μ*L of ice-cold ethanol (*Merck, Germany*) were added to the mixture obtained from the last centrifuge and it was shaken to ensure the mixing of the fluids. The mixture was centrifuged at 10000 ×g for 15 minutes and the upper part was removed completely. 1 mL of 70% ethanol was added to the lower part [19]. Then, 0.5 mL of 60% formic acid (*Sigma-Aldrich, Germany*) was added to 1 mL of the mixture obtained and it was left for 60 minutes at 150°C. The tubes were held at room temperature in order to eliminate the formic acid and approximately 1 mL of the mixture was stored at −20°C until the day of the analysis [20].

#### 3.2.2. HPLC Analysis of 8-Hydroxyguanine

The levels of 8-OHdG and deoxyguanine (dG) were measured using different wavelengths and using HPLC-UV and HPLC-ECD electrochemical detectors. Before HPLC analysis, hydrolysed DNA samples were dissolved in an appropriate solvent. The final volume was 1 mL. Then, 20 *μ*L of the final hydrolysate was injected into the HPLC-ECD (HP, HP 1049A ECD detector, Agilent 1100 modular systems, HP 1049A ECD detector, Germany). A reverse phase C18 (RP-C18) analytical column (250 mm × 4.6 mm × 4.0 *μ*m, Phenomenex, CA) was used. The mobile phase consisted of acetonitrile (*Merck, Germany*) (97 : 3, v/v) containing 0.05 M potassium phosphate (*Merck, Germany*) (pH 5.5) buffer solution with a flow rate of 1 mL per minute. The absorbance of the dG concentrate was measured at 245 nm and 8-OHdG was read electrochemically (600 mV). The amounts of dG and 8-OHdG were determined using dG and 8-OHdG (*Sigma-Aldrich, Germany*) standards. 8-OHdG7 dG106 was interpreted as a sign of DNA damage [19, 21, 22].

#### 3.2.3. Determination of COX-2 Gene Expression

First, 200.0 *μ*L of the extract obtained from the fragmented tissue was placed in a MagNA Pure Compact automatic RNA isolation device (Roche). Then, a 50.0 *μ*L RNA sample was obtained through RNA isolation using the MagNA Pure Compact RNA isolation kit (Roche).

#### 3.2.4. cDNA Synthesis

The concentration of the RNA obtained was measured. Based on the measured DNA concentration, the RNA was either diluted or undiluted to yield 15–20 ng of cDNA. Then, 10.0 *μ*L of each calibrated sample, 2.0 *μ*L of random primer, and 1.0 *μ*L of distilled water from the Transcriptor First Strand cDNA Synthesis Kit (tube number 6) were transferred into the 0.2-PCR tube. Denaturation was then conducted in the reverse-transcription PCR instrument at 65°C for 10 min. The mixture was added to the denatured RNA to form cDNA. The quantities of the substances included in the mixture used for each sample were as follows (from the Transcriptor First Strand cDNA Synthesis Kit): 4.0 *μ*L of the reaction buffer (number 2), 5.0 *μ*L of RNAase (number 3), 2.0 *μ*L of the deoxynucleotide mix (number 4), and 0.5 *μ*L of the reverse transcriptase (number 1). After the addition of 7.0 *μ*L of the mixture to the denatured RNA, the tube was placed in the reverse-transcription PCR instrument and subjected to an appropriate PCR program.

#### 3.2.5. Gene Expression Analysis

For each cDNA sample, gene expression was analyzed using the Roche LightCycler 480 II Real-Time PCR instrument (Mannheim, Germany). PCR reactions were in a final volume of 20 *μ*L: 5 *μ*L cDNA, 3 *μ*L distilled water, 10 *μ*L LightCycler 480 Probes Master (Roche Diagnostics), and 2 *μ*L primer probe set (Real-Time Ready single assay, Roche). Cycle conditions of the relative quantitative PCR (qPCR) were preincubation at 95°C for 10 min, followed by 45 amplification cycles of 95°C for 10 s, 6°C for 30 s, and 72°C for 1 s, followed by cooling at 40°C for 30 s. qPCR analysis and calculation of quantification cycle (Cq) values for Relative Quantification were performed with the LightCycler 480 software, version 1.5 (Roche Diagnostics). Relative quantitative amounts were calculated by dividing the target genes by the expression level of the reference gene. Reference gene was used for normalization of target gene expression.

### 3.3. ALT and AST Analysis

Venous blood samples were collected into tubes without anticoagulant. Serum was separated by centrifugation after clotting and stored at −80°C until it was assayed. Serum AST and ALT activities were measured spectrophotometrically as liver function tests and LDH activity was measured as a marker of tissue injury, using a Cobas 8000 (Roche) autoanalyzer with commercially available kits (Roche Diagnostics, GmBH, Mannheim, Germany).

#### 3.3.1. ALT Analysis

Quantitative determination of the serum ALT (Alanine Aminotransferase) was studied by spectrophotometric method using Roche Cobas 8000 autoanalyser. According to the International Federation of Clinical Chemistry (IFCC), pyridoxal 5′-phosphate method catalyzes the reaction between 3,4 ALT L-alanine and 2-oxoglutarate. The pyruvate formed is reduced by NADH with a reaction catalyzed by lactate dehydrogenase (LDH) in which L-lactate and NAD^+^ are formed. Pyridoxal phosphate functions as a coenzyme in the amino transfer reaction. It enables enzyme activation to be complete. L-Alanine + 2-oxoglutarate →  ^(ALT)^pyruvate + L-glutamate. Pyruvate + NADH + H^+^ → ^(LDH)^L-lactate + NAD^+^. Rate of the NADH oxidization is proportional to the ALT activity.

#### 3.3.2. AST Analysis

Quantitative determination of the serum AST (Aspartate Aminotransferase) was studied by spectrophotometric method using Roche Cobas 8000 autoanalyser. According to the International Federation of Clinical Chemistry (IFCC), pyridoxal 5′-phosphate method catalyzes the transfer of an amino acid group between L-aspartate and 2-oxoglutarate in order to form 3,4 AST oxaloacetate and L-glutamate in the sample. Then oxaloacetate reacts with NADH in the presence of malate dehydrogenase (MDH) in order to provide formation of NAD^+^. Pyridoxal phosphate functions as a coenzyme in the amino transfer reaction. L-Aspartate + 2-oxoglutarate → ^(AST)^oxaloacetate + L-glutamate. Oxaloacetate + NADH +  H^+^  → ^(MDH)^L-malate + NAD^+^. Rate of the NADH oxidization is proportional to the AST activity.

### 3.4. Statistical Analysis

Data obtained from the experiments were expressed as “mean ± SEM” (*x*  ± SEM). Significance of the differences between the groups was determined using one-way ANOVA test followed by Fisher's post hoc LSD (least significant differences). All the statistical processes were carried out with “SPSS for Windows, 18.0,” statistical software and *p* < 0.05 values were considered as significant.

## 4. Results

**Figure 1:**
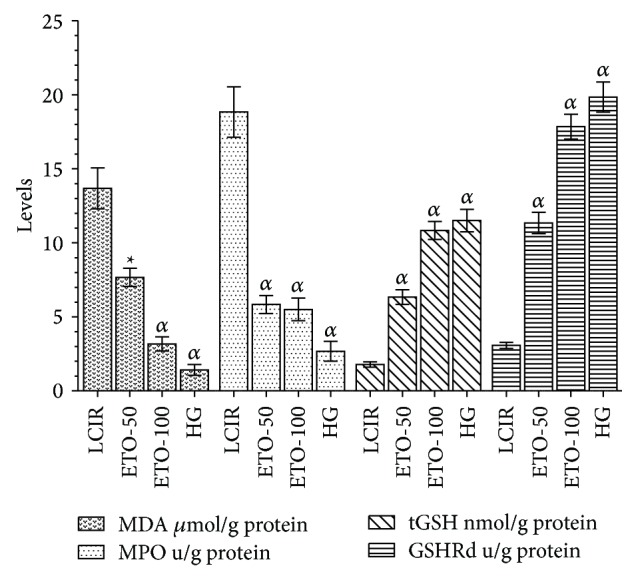
The effects of etoricoxib on MDA, MPO, tGSH, and GSHRd levels in rats (ETO-50: etoricoxib-50 mg group, ETO-100: etoricoxib-100 mg group, LIRC: liver ischemia reperfusion control group, and HG: healthy group, *N* = 6).

MDA levels in liver tissues of the KCR, ETO-50, ETO-100, and SG groups were, respectively, 13.7 ± 2.6, 7.7 ± 1.2 (*p* < 0.001), 5.2 ± 0.19 (*p* < 0.0001), and 3.2 ± 0.9 (*p* < 0.0001) *μ*mol/gr protein, and especially MPO activities of the groups were in order of 18.8 ± 3.2, 5.8 ± 1.16 (*p* < 0.0001), 3.8 ± 1.16 (*p* < 0.0001), and 1.8 ± 0.56 (*p* < 0.0001) u/g protein as seen from Figure 1. While tGSH level of ETO-50 group was 6.3 ± 1 (*p* < 0.0001) nmol/g protein and GSHRd level was 11.3 ± 1.33 (*p* < 0.0001) u/g protein, tGSH level of ETO-100 group was 10.8 ± 1.16 (*p* < 0.0001) nmol/g protein and GSHRd level was determined as 17.8 ± 1.56 (*p* < 0.0001) u/g protein. tGSH and GSHRd levels of the KCR group were, respectively, 1.8 ± 0.35 nmol/g protein and 3.1 ± 0.36 u/g of protein. In the SG group, tGSH and GSHRd levels were calculated as 11.5 ± 1.5 (*p* < 0.0001) nmol/g protein and 8.19 ± 1.83 (*p* < 0.0001) u/g protein. So etoricoxib statistically significantly prevented increase of the MDA and MPO levels and decrease of the tGSH and GSHRd levels at 50 and 100 mg/kg doses compared to LIRC group in the liver tissue subjected to I/R.

**Figure 2:**
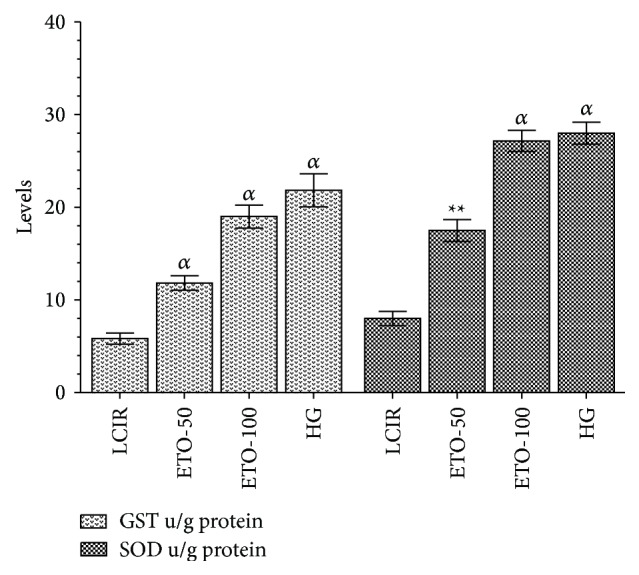
The effects of etoricoxib on GST and SOD levels in rats (ETO-50: etoricoxib-50 mg group, ETO-100: etoricoxib-100 mg group, LIRC: liver ischemia reperfusion control group, and HG: healthy group, *N* = 6).

As seen from Figure 2, etoricoxib at 50 and 100 mg/kg doses inhibited reduction of the antioxidants such as GST and SOD in the liver I/R damage. GST activities in the liver tissues of the KCR, ETO-50, ETO-100, and SG groups were found in order of 5.8 ± 1.16, 11.8 ± 1.5 (*p* < 0.0001), 19 ± 2.3 (*p* < 0.0001), and 21.8 ± 3.5 (*p* < 0.0001) u/g protein. Again, in this graphic, for these groups, SOD activity is observed as 8.0 ± 1.6, 17.5 ± 2.5 (*p* < 0.001) 2.27 ± 2.16 (*p* < 0.0001), and 28.0 ± 2.3 (*p* < 0.0001) u/g protein, respectively.

**Figure 3:**
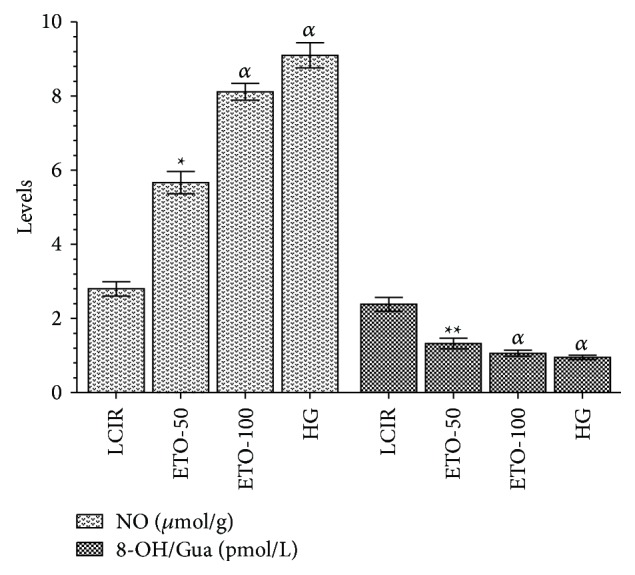
The effects of etoricoxib on NO and 8-OHdG levels in rats (ETO-50: etoricoxib-50 mg group, ETO-100: etoricoxib-100 mg group, LIRC: liver ischemia reperfusion control group, and HG: healthy group, *N* = 6).

As Figure 3 to be understood, 100 mg/kg dose of etoricoxib administered group's NO amounts (8.1 ± 0.41, *μ*mol/g protein, *p* < 0.0001) in liver tissue was found higher than 50 mg/kg dose etoricoxib administered group's NO amounts (5.7 ± 0.56 *μ*mol/g protein, *p* < 0.01). So, amount of NO in the liver of rats in the ETO 100 group was higher than in the ETO 50 group. That is, etoricoxib administered at 100 mg/kg dose is seen to more significantly inhibit decrease of NO than the group which received 50 mg/kg dose.

Etoricoxib at the same doses also suppressed increase of the amount of 8-OH/Gua which is a product of DNA damage. However, amount of 8-OH/Gua was higher at 100 mg/kg dose than at 50 mg/kg. Likewise, ETO-100 group's 8-OH/Gua amount (1.06 ± 0.15 *p* < 0.0001 pmol/L) was significantly decreased compared with the ETO-50 group (1.3 ± 0.27 pmol/L, *p* < 0.01). NO and 8-OH/Gua levels in KCR group were 2.8 ± 0.36 *μ*mol/g protein and 2.4 ± 0.38 pmol/L, while these values were 9.1 ± 0.6 *μ*mol/g protein and 0.95 ± 0.1 pmol/L in group SG.

**Figure 4:**
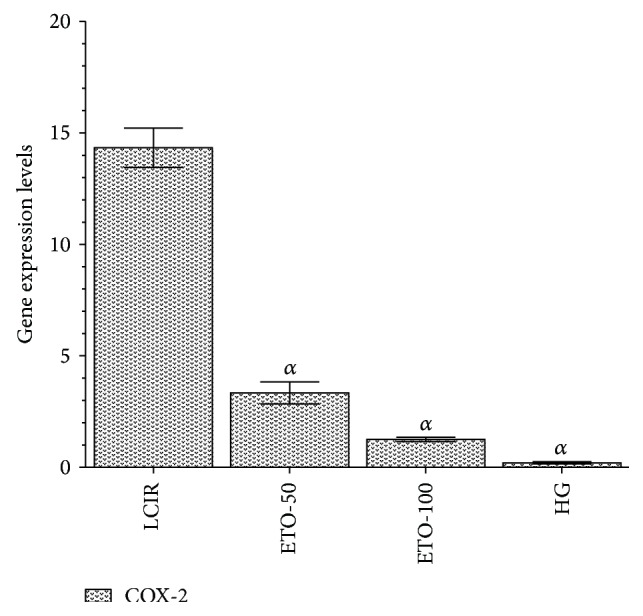
The effects of etoricoxib on COX-2 gene expression in rats (ETO-50: etoricoxib-50 mg group, ETO-100: etoricoxib-100 mg group, LIRC: liver ischemia reperfusion control group, and HG: healthy group, *N* = 6).

Etoricoxib 50 and 100 mg/kg doses significantly decreased COX-2 gene expression which was increased by ischemia and reperfusion. COX-2 gene expression levels in KCR, ETO-50, ETO-100, and SG groups were found, respectively, as 14.3 ± 1.71 and 3.3 ± 0.93 (*p* < 0.0001), 1.25 ± 0.2 (*p* < 0.0001), and 0.2 ± 0.1 u/g protein in liver tissues (Figure 4).

**Figure 5:**
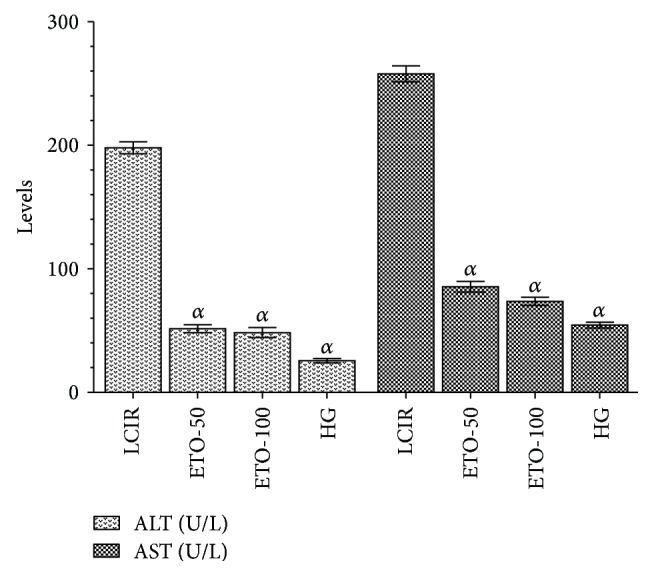
The effects of etoricoxib on ALT and AST levels in rats (ETO-50: etoricoxib-50 mg group, ETO-100: etoricoxib-100 mg group, LIRC: liver ischemia reperfusion control group, and HG: healthy group, *N* = 6).

While ALT levels of the groups ETO-50 and ETO-100 were calculated as 51.5 ± 5.6 (*p* < 0.0001) and 48.3 ± 8 (*p* < 0.0001), AST levels of these groups were 85.5 ± 7.8 (*p* < 0.0001) and 73.4 ± 6.3 (*p* < 0.0001), respectively. ALT and AST levels for SG group were calculated as 25.7 ± 3.3 (*p* < 0.0001) and 54.5 ± 4.8 (*p* < 0.0001) and again for KCR group were 198 ± 9.6 and 258 ± 12.1. So ALT and AST activities were found to be statistically significantly higher in the liver tissue of LIRC group than in the ETO-50, ETO-100, and HG groups. Difference between the ETO-50 and ETO-100 in terms of the ALT and AST activities was not statistically significant (Figure 5).

## 5. Discussion

In this study, effect of etoricoxib on the liver damage induced by I/R was investigated. We evaluated whether I/R created damage in the liver tissue using measurement of oxidant/antioxidant parameters, COX-2 gene expression, and serum levels of ALT and AST. As is known, toxicity on the tissue may be of biochemical, functional, or structural nature. Severity of the biochemical toxic impact has been shown to be proportional to the severity of histopathological findings [23]. Our experimental results showed that levels of oxidant/antioxidant parameters such as MDA, MPO, tGSH, GSHRd, GST, SOD, and NO that we use in determination of I/R-related biochemical toxicity changed in favour of oxidants in LIRC group. Oxidant/antioxidant balance is maintained by superiority of antioxidants. Several aggressive factors that may lead to tissue damage provide oxidant/antioxidant balance to impair in favour of oxidants, termed as oxidative stress. That is, occurrence of tissue damage is evaluated through oxidant/antioxidant balance [6]. Etoricoxib changes oxidant/antioxidant balance in favour of antioxidants in the liver tissue with I/R induced, showing that our results were consistent with the literature. In literature screening, there is not a study about protective effect of etoricoxib which has antioxidant and anti-inflammatory features on the liver I/R damage. However, recent studies reported that etoricoxib protects renal tissue against I/R damage by preventing increase of MDA and MPO levels and decrease of the amount of tGSH [3]. Again, Yapca et al. reported that etoricoxib decreased MDA and MPO levels which increased in the ovarian I/R damage and inhibited reduction of tGSH levels [10]. Besides decreasing MDA and MPO and increasing tGSH, etoricoxib administration after I/R induction prevented decrease of the parameters such as GSHRd, GST, and SOD. GSHRd, GST, and SOD are enzymatic antioxidant molecules, protecting cells against the damage by free oxygen radicals [24]. Levels of these enzymatic antioxidants were higher in the etoricoxib group than LIRC group, supported by the results of a study conducted by Kanwar et al. [25]. However, there are some results in the literature which are not consistent with ours; Maheshwari et al. reported that etoricoxib decreased the amount of NO in the cerebral I/R damage [26]. NO is accepted as an oxidant since it has one unpaired electron. However, NO has been reported to have also beneficial effects on the regulation of vascular tonus [27–29]. Again it has been suggested in the studies which support our results that amount of NO decreased in the hepatic I/R damage; thus NO may be helpful in treatment of the liver I/R damage [30, 31].

Excessively produced free oxygen radicals may react with macromolecules in the cells and lead to severe cellular damage including lipid peroxidation, oxidative modification of proteins, and DNA oxidation [6]. Amount of 8-OH/Gua was significantly higher in the LIRC group, in which oxidant parameters were high, than the etoricoxib group. Also it has been demonstrated in the literature that amount of 8-OH/Gua is elevated in the liver I/R damage with increased oxidant values than in the healthy tissues [7]. Polat et al. reported that amount of 8-OH/Gua increases proportionally to the oxidants [32]. Therefore, our results can be assessed as consistent with information from the literature.

Again, in our study, we found that COX-2 gene expression slowed down in the etoricoxib group compared to the LIRC group. COX-2 is rather low in the healthy tissues, while it is known to markedly increase in the inflamed tissues [7], demonstrating I/R damage is limited by inflammatory response at the etoricoxib doses. In their study, Suleyman et al. supported with immunohistochemical studies that protective effect of etoricoxib on the I/R damage resulted from its inhibitory activity on COX-2 [3]. Yapca et al. argued that etoricoxib protected the ovarian tissue against I/R damage. It was reported in the same study that etoricoxib inhibited increase of COX-2 activity in the ovarian tissue [10].

Blood ALT and AST activities which are measured to evaluate protective effect of etoricoxib in the liver I/R damage are the most commonly used parameters in evaluation of the hepatic function [33]. Tuncer et al. reported that levels of ALT and AST are elevated during the hepatic I/R damage [34]. Also ALT and AST have been experimentally shown to significantly increase in the hepatic oxidative tissue damage caused by I/R [7]. Free oxygen radicals whose production is stimulated in the liver I/R damage are accused of the increase in ALT and AST activities [33]. ALT and AST activities were found to be significantly lower and very close to the baseline values in the etoricoxib group than the LIRC group. This can be considered to indicate that liver functions in rats were much more different in the etoricoxib group than in the LIRC group. In conclusion, etoricoxib prevented the liver oxidative damage due to I/R. Oxidant/antioxidant balance changed in favour of oxidants in the LIRC and in favour of antioxidants in the etoricoxib group. In addition, etoricoxib improved hepatic dysfunctions caused by I/R. This information suggests that etoricoxib can be beneficial in prevention of the damage which may emerge in the clinical I/R process.
